# A cross-subject decoding algorithm for patients with disorder of consciousness based on P300 brain computer interface

**DOI:** 10.3389/fnins.2023.1167125

**Published:** 2023-07-20

**Authors:** Fei Wang, Yinxing Wan, Zhuorong Li, Feifei Qi, Jingcong Li

**Affiliations:** ^1^School of Software, South China Normal University, Guangzhou, China; ^2^Pazhou Lab, Guangzhou, China; ^3^School of Internet Finance and Information Engineering, Guangdong University of Finance, Guangzhou, China

**Keywords:** brain computer interface, P300, disorder of consciousness, cross-subject, EEG

## Abstract

**Background:**

Brain computer interface (BCI) technology may provide a new way of communication for some patients with disorder of consciousness (DOC), which can directly connect the brain and external devices. However, the DOC patients’ EEG differ significantly from that of the normal person and are difficult to collected, the decoding algorithm currently only is trained based on a small amount of the patient’s own data and performs poorly.

**Methods:**

In this study, a decoding algorithm called WD-ADSTCN based on domain adaptation is proposed to improve the DOC patients’ P300 signal detection. We used the Wasserstein distance to filter the normal population data to increase the training data. Furthermore, an adversarial approach is adopted to resolve the differences between the normal and patient data.

**Results:**

The results showed that in the cross-subject P300 detection of DOC patients, 7 of 11 patients achieved an average accuracy of over 70%. Furthermore, their clinical diagnosis changed and CRS-R scores improved three months after the experiment.

**Conclusion:**

These results demonstrated that the proposed method could be employed in the P300 BCI system for the DOC patients, which has important implications for the clinical diagnosis and prognosis of these patients.

## 1. Introduction

Coma, unresponsive wakefulness syndrome (UWS), also known as vegetative state (*VS*), minimally conscious state (MCS), and emergence from MCS are all considered disorders of consciousness (DOC) (Xiao et al., 2016). The patients with DOC are usually assessed clinically by a doctor based on a behavior scale [e.g., the Glasgow Coma Scale (GCS), the Coma Recovery Scale-Revised (CRS-R)], which is based on the doctor’s empirical judgment and highly subjective (Sternbach, 2000; Kalmar and Giacino, 2005). In addition, the patients lack adequate and stable behavioral responsiveness, which leads to an extremely high percentage of vegetative state misdiagnoses by the scale’s behavioral-based approach (Van Erp et al., 2014; Johnson and Lazaridis, 2018). In previous research in 2020, the researchers analyzed 137 patients with long-term DOC by using the CRS-R scale. The results showed a misdiagnosis rate of 24.7% in patients with MCS assessed by a single CRS-R scale and over 38% in patients with MCS assessed by repeated CRS-R assessments (Wang et al., 2020). Therefore, accurate clinical diagnosis of people with DOC is challenging.

To resolve these issues, several researchers have considered utilizing brain-computer interfaces (BCI) to assess the DOC patients’ state. This approach can directly detect the brain’s response to an external stimulus without requiring behavioral or verbal expression from the patients. In an early study (Cruse et al., 2011), a novel EEG experiment incorporating motor imagery was developed to identify command-following in the absence of obvious behavior. While assessing 16 *VS* patients, 3 of them could reliably and repeatedly produce suitable EEG responses to two different commands. This indicated that EEG technique can detect awareness in some *VS* patients. In recent years, researchers have worked more on the BCI system based on P300 and steady-state evoked potential (SSVEP) compared to other paradigms. These systems primarily utilize external stimuli to generate P300 signals or SSVEP signals from DOC patients, and then decode the signals to accomplish the assessment or communication with the patients. A visual hybrid BCI system incorporating P300 and SSVEP responses was developed to assess awareness in severely brain injured individuals by Pan et al. (2014). It could determine which photo the patient focuses on. Among 7 patients (4 *VS* and 3 MCS patients), 2 (one *VS* and one MCS patients) were able to selectively attend to their own or unfamiliar photos. Huang et al. (2021) applied a hybrid asynchronous BCI system that presents DOC patients with a new way to communicate. The patients were directed to pay attention to the squares bearing the words “Yes” and “No.” Three (MCS patients) of 7 patients (3 *VS* and 4 MCS patients) could utilize their hybrid asynchronous BCI system to communicate, which demonstrates that both the P300-only and SSVEP-only systems underperformed the hybrid asynchronous BCI system. Xiao et al. (2022) developed an innovative audiovisual BCI system to model the evaluation of sound localization in CRS-R. Among 18 patients, 11 patients showed sound localization in the BCI system and 4 in CRS-R assessment. More and more efficient BCI systems begin to be applied to the auxiliary diagnosis and evaluation of the DOC patients.

While P300 is easier to stimulate compared to SSVEP, P300 signal can be stimulated in many ways, including using visual and auditory (Li et al., 2019). These make it more widely used in DOC patients. Therefore, a decoding algorithm that can accurately detect the patient’s P300 signal may improve the diagnostic accuracy of the patient’s current status. However, compared to healthy individuals, DOC patients have less pronounced P300 features and greater differences (Li et al., 2022). Meanwhile, DOC patients are easily fatigued, which makes data collection challenging (Wang F. et al., 2019). Most current systems basically adopt the intra-subject decoding (Murovec et al., 2020). These approaches require users to first undergo a period of calibration to train a reliable model, which largely affects the widespread adoption of BCI systems. Therefore, it would be beneficial to design an excellent cross-subject decoding algorithm for BCI on DOC patients.

In current researches, the cross-subject P300 decoding algorithms are based on healthy human data for analyzing, and convolutional neural network (CNN) is one of the most efficient decoding algorithms (Mijani et al., 2020). In 2010, Cecotti and Graser (2010) proposed a CNN-based P300 detection method, which won the 3rd BCI competition. The approach sequentially extracted channel features and temporal features using a four-layer CNN architecture, which showed CNNs could capture spatial features and potential sequence dependencies from EEG signals. However, while CNNs have increased detection precision to previously unheard-of levels, there are still obstacles to this approach. Its network accuracy depends on the training data’s quantity and quality (Wang et al., 2021). Furthermore, due to the high cost of time and labor, the P300 task commonly has a little amount of high-quality data. EEGNet (Lawhern et al., 2018) was proposed as a generalized deep network, which was implemented by deeply separable convolution, and it produced satisfactory results in various EEG detections. By using original EEG information, the network can perform sequence learning directly and then generalize the acquired dependencies in the spatial domain. Abibullaev et al. (2022) used the leave-one-subject cross-validation method to test the cross-subject capability of several CNNs on four publicly available datasets. Their results indicated that EEGNet and ShallowConvNet (Schirrmeister et al., 2017) had better performance. Alvarado-Gonzalez et al. (2021) proposed a simple network, SepConv1D, which consists of a depth-separable one-dimensional convolutional layer and a fully connected Sigmoid classification neuron. And only four filters and a minimal set of parameters make up SepConv1D’s convolutional layer, but its performance is competitive. Although the current P300 detection methods have achieved good results in normal individuals, these may be not suitable for DOC patients. DOC patients may not be able to process information effectively, or their level of consciousness may be reduced, resulting in delayed or weakened P300 signals and decreased occurrence rates (Zhang et al., 2017). Furthermore, as there are differences in the P300 signal between different DOC patients, it is difficult to achieve good results when training with other patients’ data. Due to the difficulty of collecting a large amount of data from DOC patients who are easily fatigued and difficult to control, studying cross-subject algorithms for DOC patients can be beneficial for clinical application.

In this study, we proposed a domain adaptation-based cross-subject P300 decoding algorithm for DOC patients. This method used healthy subjects’ data to train the model, and then used an adversarial approach to adapt the network. The experiment results show our approach achieved the same level of cross-subject accuracy in DOC patients as the traditional intra-subject approach.

## 2. Materials and methods

### 2.1. Subjects

This study involved data from 19 subjects (including 11 DOC patients and 8 healthy subjects). Prior to the experiment, all subjects (or patients’ relatives) provided written informed consent that they all signed. All data were acquired with 36-conductor Greentech electrode caps and a SynAmps2 amplifier from Neuroscan. The sampling rate of the amplifier was set at 250 Hz. 10 channels of EEG data (O1, Oz, O2, P7, P3, Pz, P4, P8, Fz, and Cz) were acquired from each subject wearing electrode caps according to the Extended International 10–20 System standard. To ensure signal quality during collecting data, all electrodes’ scalp contact impedances were kept below 5 kΩ. Healthy subjects need to be between 18–55 years old, right-handed, and have normal or corrected-to-normal vision. Eight healthy subjects were males between the ages of 23 and 33 (mean 26.38 years). For patients, they are between 18 and 70 years old, with a disease course of no more than 1 year, right-handed, and have no history of diseases that cause perceptual impairments such as visual or auditory impairments. They also have no history of neurological or psychiatric diseases, or severe psychiatric symptoms. In this experiment, the diagnosis of each patient was evaluated using the CRS-R, which is the gold standard for clinical behavioral diagnosis, to assess various aspects such as auditory, visual, motor, language, response, and level of consciousness. Each patient’s CRS-R score was evaluated by the same doctor. In this study, a total of two CRS-R assessment results were obtained: one before the start of the experiment and the other 3 months after the end of the experiment. When evaluating the patient’s CRS-R score, the doctor observed the patient’s best state and chose one day to score multiple times, taking the best score as the final result. Table 1 shows the patients’ details.

**Table 1:** The information and CRS-R scores for all DOC patients.

Patient	Age (years)	Gender	Etiology	Time since injury (months)	Before experiment		After 3 months	
					CRS-R score (subscores)	Diagnosis	CRS-R score (subscores)	Diagnosis
P1	29	M	ABI	8.5	4 (1-0-1-0-0-2)	UWS	4 (1-0-1-0-0-2)	UWS
P2	37	M	ABI	2	5 (0-0-2-1-0-2)	UWS	5 (0-0-2-1-0-2)	UWS
P3	38	M	TBI	1	7 (1-1-2-1-0-2)	UWS	7 (1-1-2-1-0-2)	UWS
P4	33	M	TBI	2	7 (1-0-2-2-0-2)	UWS	7 (1-0-2-2-0-2)	UWS
P5	48	M	ABI	4	7 (1-1-2-1-0-2)	UWS	18 (4-5-5-1-1-2)	MCS+
P6	19	M	CVD	2	7 (1-1-2-1-0-2)	UWS	15 (4-5-2-2-0-2)	MCS+
P7	38	M	TBI	2	10 (1-3-3-1-0-2)	MCS-	19 (3-5-6-1-1-3)	MCS+
P8	44	M	CVD	2.5	9 (1-3-2-1-0-2)	MCS-	20 (4-5-6-2-1-2)	MCS+
P9	17	M	TBI	2	8 (1-1-3-1-0-2)	MCS-	18 (4-5-3-1-2-3)	MCS+
P10	46	F	TBI	1.5	7 (1-0-3-1-0-2)	MCS-	19 (3-5-6-2-1-2)	MCS+
P11	46	M	CVD	2	9 (1-1-4-1-0-2)	MCS-	20 (4-5-6-2-1-2)	MCS+

ABI, anoxic brain injury; TBI, traumatic brain injury; CVD, cerebrovascular disease.

### 2.2. Experimental paradigm

**Figure 1:**
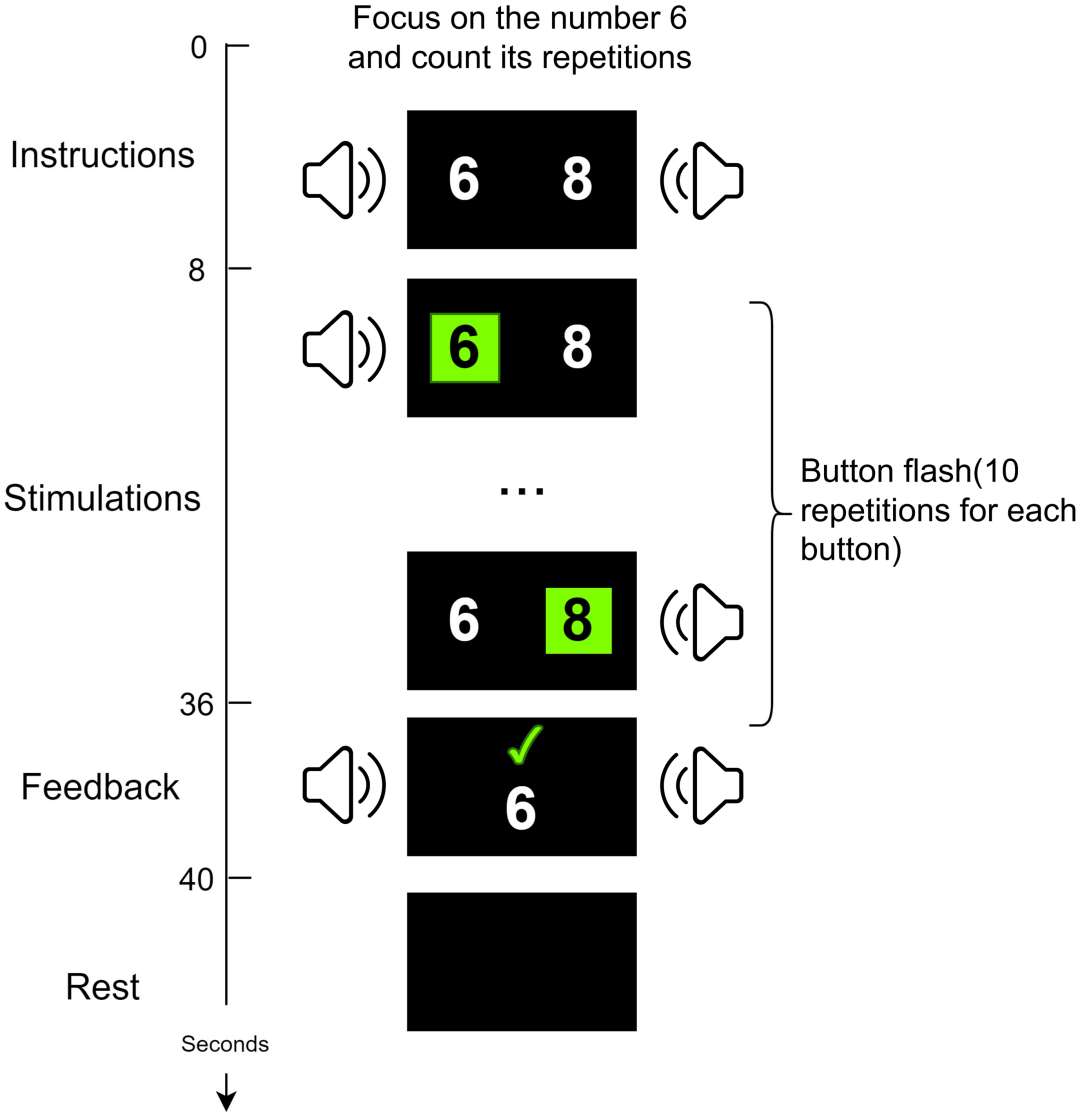
The P300-based audio-visual BCI system.

The data in this study were collected through a P300-based audio-visual BCI system (Wang et al., 2015; Pan et al., 2020). Based on the patient’s condition, data collection was conducted for 1–2 days each week, with 1–2 blocks collected per day. The BCI experiment for each patient lasted for approximately 2 weeks. As shown in Figure 1, two random numbers ranging from 0–9 (e.g., 6, 8) appear on each side of the screen. Two speakers are placed at the side and rear of each side of the display. First, the user was introduced to his task through a 6s Chinese audiovisual instruction. And then, the two digital buttons flash alternately between black and green. At the same time, the speaker on the same side as the flashing digit presents the corresponding voice digit. Finally, the results are displayed on the monitor and voice feedback is given. At the end of the experiment, healthy subjects are given a 2s rest period. In contrast, for DOC patients, there is at least a 10s rest period. Since the patients are easily fatigued and unable to persist in acquiring data from multiple blocks consecutively, the acquisition of patient data needs to be done in multiple sessions based on their physical and mental conditions, as suggested by the medical staff.

**Figure 2:**
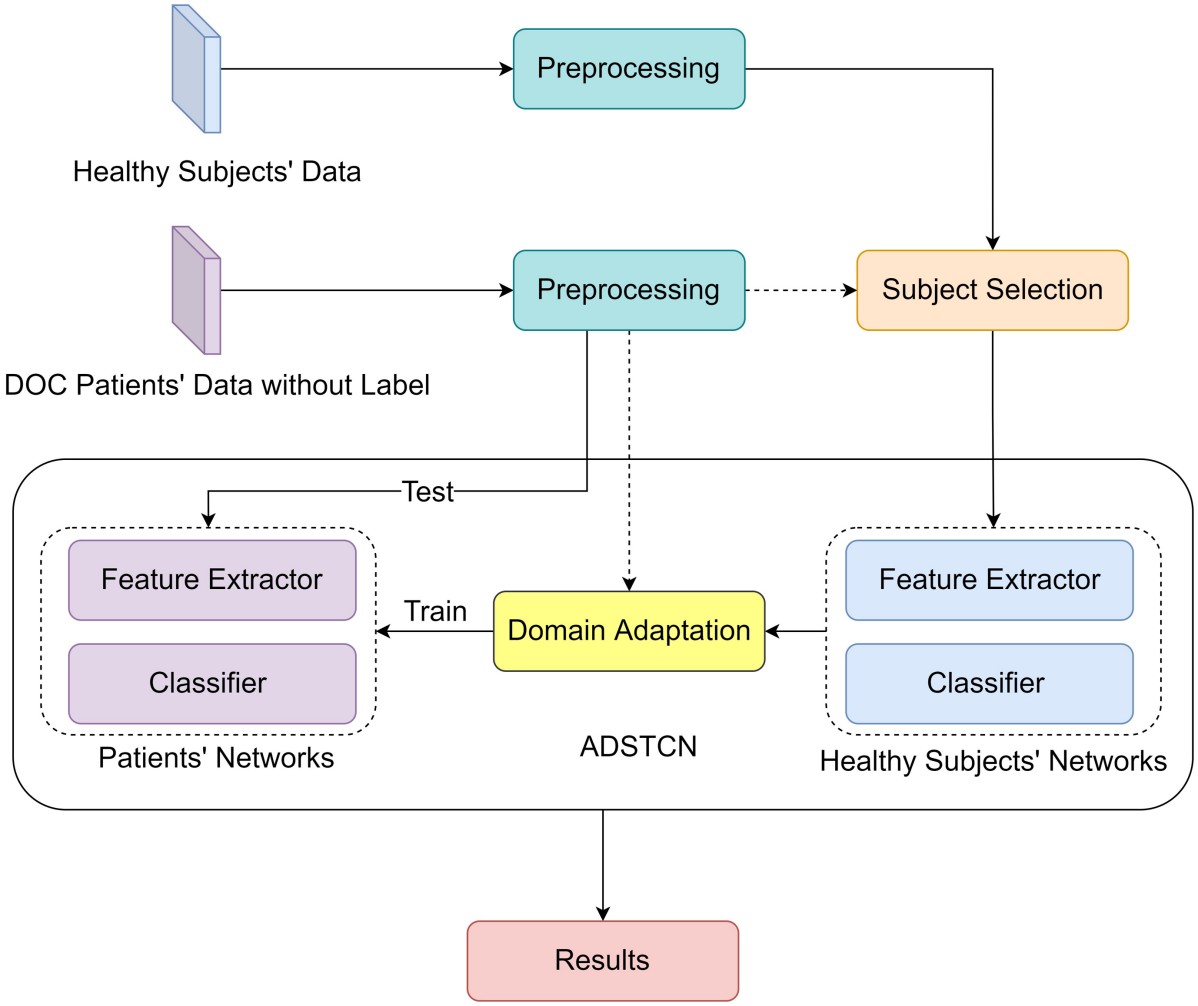
The framework of the proposed WD-ADSTCN.

### 2.3. Algorithm description

The experiments were performed on a single PC with Linux Ubuntu 20.04.3 LTS, an Intel(R) Core (TM) i9-12900 K CPU @ 5.20 GHz, 96 GB in RAM, and an NVIDIA GeForce RTX 3090 GPU and 24 GB of RAM. The networks were implemented in Pytorch 1.12.0 (Paszke et al., 2019) as backend. Figure 2 reports the Deep Learning algorithm pipeline. The raw EEGs of patients and healthy subjects are collected after data preprocessing, and then the data of healthy subjects are selected by Wasserstein distance (WD) (Vallender, 1974) for training. And then based on the Adversarial Discriminative Domain Adaptation (ADDA) (Tzeng et al., 2017) algorithm, we proposed an WD-Adversarial Discriminative Spatio-Temporal Convolution Network (WD-ADSTCN) to adapt the feature extractor and classifier to obtain the final results.

**Figure 3:**
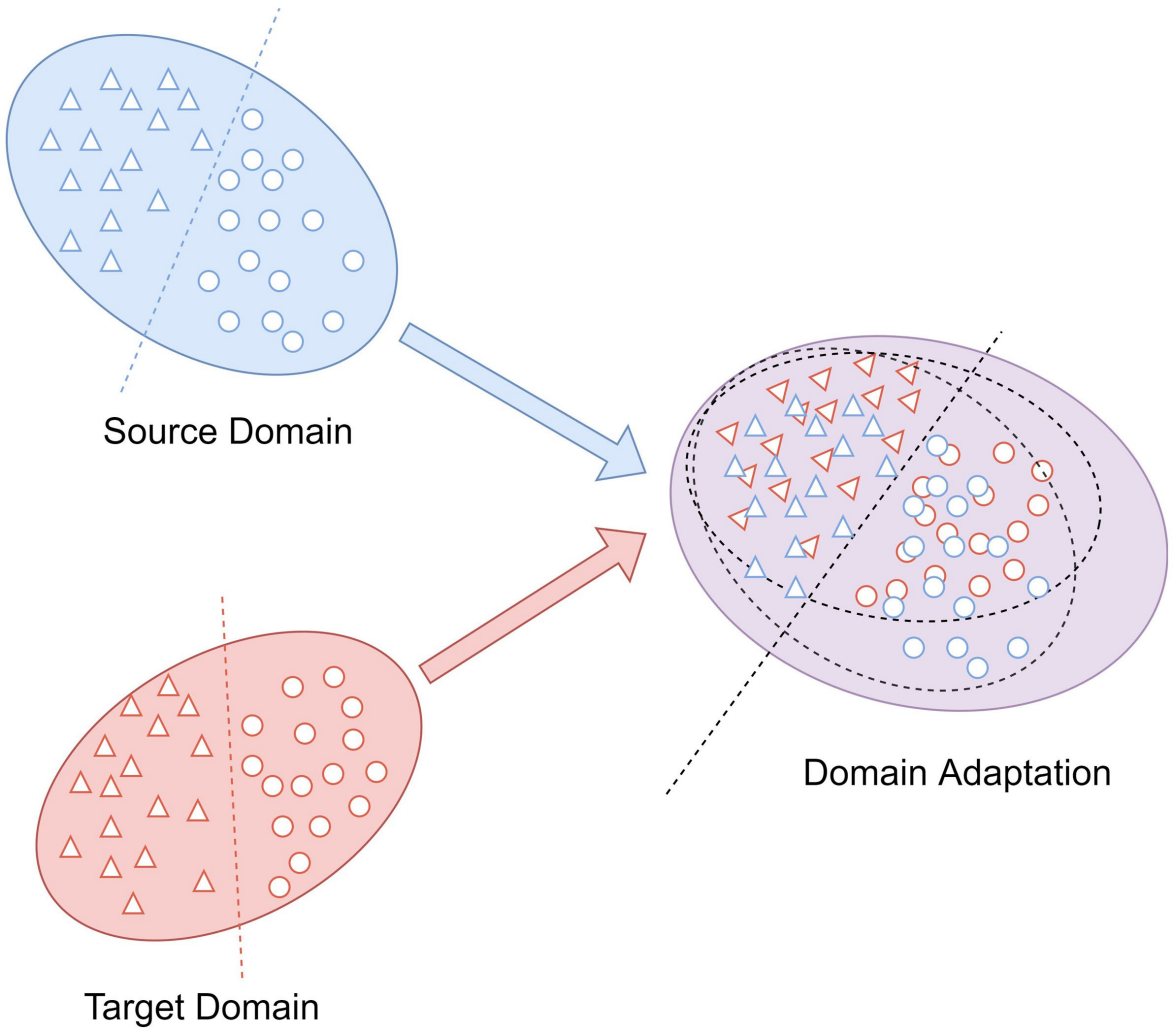
The main idea of domain adaptation.

#### 2.3.1. Preprocessing

In this study, all channel EEG data within 1000ms after the subject was stimulated were extracted as samples, and baseline correction was performed using the 100ms EEG data before stimulation, followed by 0.1–20 Hz band-pass filtering to filter out signal noise that was not in this frequency interval.

#### 2.3.2. Subject selection

Since the P300 signal of DOC patients is significantly different from that of healthy people, the P300 wave of patients is smaller and difficult to identify (Li et al., 2015). Therefore, the inclusion of some healthy subjects in the training may have side effects on 0the results. The subject selection method adopted in this study centers on the difference between the two distributions by metrics. In the current research, WD is somewhat superior to other metrics such as Jensen-Shannon Divergence (Menéndez et al., 1997), which are defined as follows:


$$W\left(p_{s},p_{t}\right)=inf_{\gamma \in \Pi \left(p_{s},p_{t}\right)}E_{\left(x,y\right)\in \gamma }\left[∥x-y∥\right]$$

where $p_{s}$ represents the source domain data distribution (healthy subjects data distribution) and $p_{t}$ represents the target domain data distribution (DOC patients’ data distribution). $\prod \left(p_{s},p_{t}\right)$ denotes a set of joint distributions, which is the collection of all the possible joint probability distributions between $p_{s}$ and $p_{t}$ .

#### 2.3.3. Domain adaptation and classification

As an integral part in transfer learning, domain adaptation is commonly applied to eliminate the differences in the feature distributions between different domains (Ren et al., 2022). The aim is to map the data into a feature space with distinct distributions for the source and target domains such that they are as close to one another as possible (Wang Z. et al., 2019). As shown in Figure 3, the source domain and the target domain with the same label space, but because of their different distributions, we cannot take the trained classifier in the source domain and utilize it to the classification of the target domain samples directly. After the domain adaptation, the trained classifier by source domain can also be employed to target domain and obtain the desired results.

**Figure 4:**
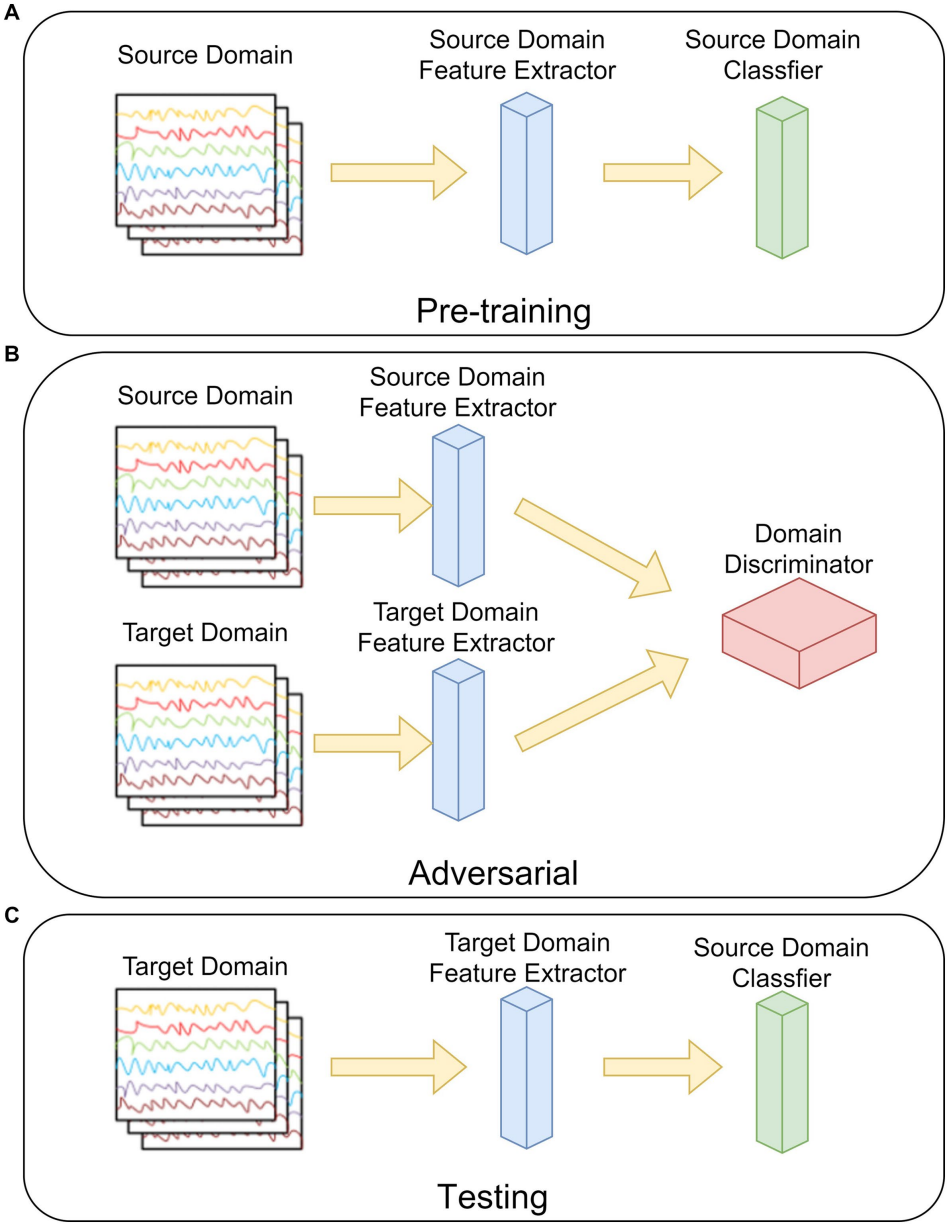
The steps of the ADSTCN network. **(A)** Pre-training. **(B)** Adversarial. **(C)** Testing.

The ADSTN algorithm will be adopted in this study. Its main structure is shown in Figure 4, which is mainly divided into three parts: pre-training, adversarial, and testing.

**Figure 5:**
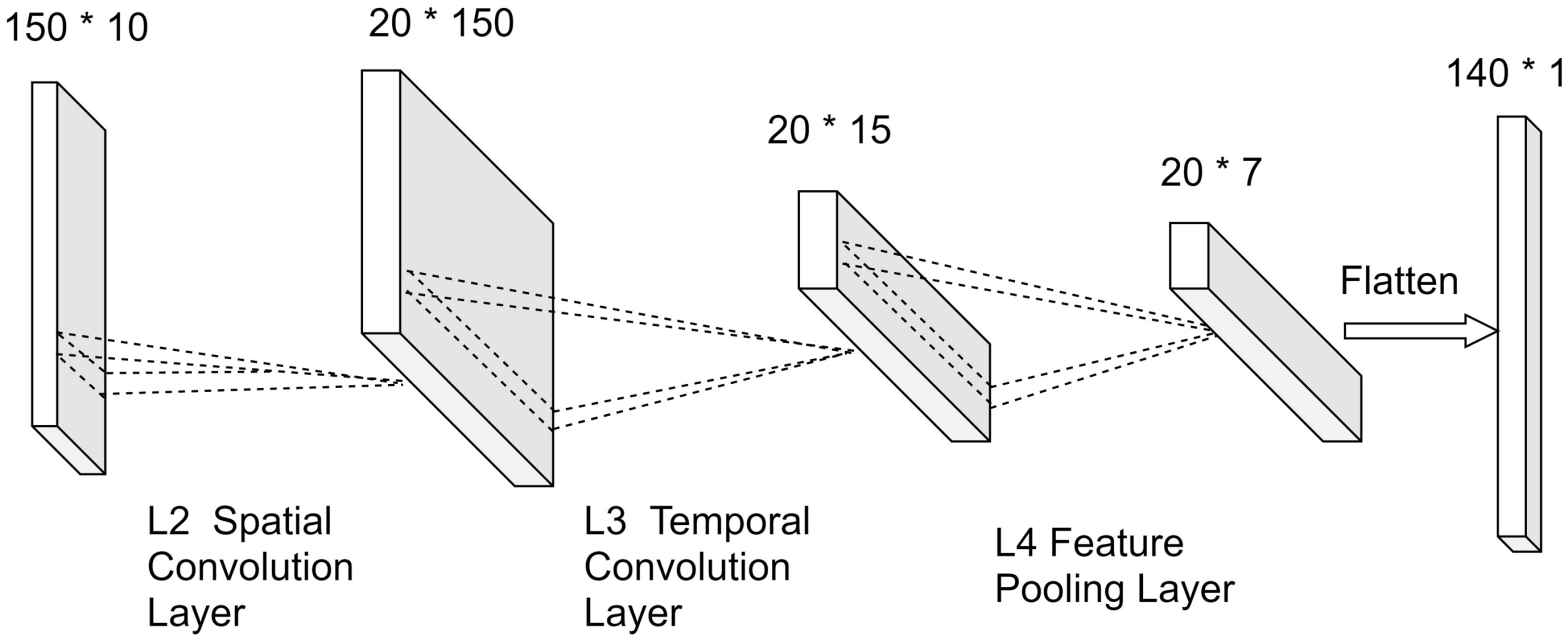
The structure of the feature extractor in the STCNN.

As in Figure 4A, in the pre-training phase, the feature extractor and classifier are trained by the source domain data. The loss of the network (cross-entropy loss) at this time can be noted as:


$$\underset{M_{s},C}{\min}L_{cls}\left(X_{s},Y_{s}\right)=-E_{\left(x_{s},y_{s}\right)\sim \left(X_{s},Y_{s}\right)}\overset{K}{\underset{k=1}{\sum }}1_{\left[k=y_{s}\right]}\log C\left(M_{s}\left(x_{s}\right)\right)$$

where $X_{s}$ is the set of all samples in source domain, $Y_{s}$ is the set of all labels in source domain, $M_{s}\left(x_{s}\right)\;$ is the extracted features from source domain feature extractor, and C(x) is the output of the classifier.

Figure 4B, before adversarial, the feature extractor weights in the target domain are initialized to be the same as the source domain feature extractor after pre-training, and then the target domain data distribution after mapping is gradually approximated to the source mapping data distribution by loss gradient transfer, so that the source domain and the target domain cannot be distinguished by the domain discriminator. The loss in the discriminator can be written as:


$$\begin{array}{l} \underset{D}{\min}L_{adv_{D}}\left(X_{s},X_{t},M_{s},M_{t}\right)=-E_{x_{s}\sim X_{s}}\left[\log D\left(M_{s}\left(x_{s}\right)\right)\right]\\ \;\;\;\;\;\;\;\;\;\;\;\;\;\;\;\;\;\;\;\;\;\;\;\;\;\;\;\;\;\;\;\;\;\;\;\;\;\;\;\;\;\;\;\;\;\;\;\;\;\;\;\;\;\;\;\;\;\;\;\;\;\;\;\;\;\;\;\;\;-E_{x_{t}\sim X_{t}}\left[\log\left(1-D\left(M_{t}\left(x_{t}\right)\right)\right)\right] \end{array}$$

where $X_{t}$ is the set of all target domain samples, $M_{t}$ is the set of target domain features, and D(x) is the output of the domain discriminator. The prediction loss of the target domain after domain adaptation can be written as:


$$\underset{M_{s},M_{t}}{\min}L_{cls}\left(X_{s},X_{t}\mathrm{,D}\right)=-E_{x_{t}\sim X_{t}}\left[\log D\left(M_{t}\left(x_{t}\right)\right)\right]$$

Figure 4C, testing the target domain feature extractor with the source domain classifier by using the target domain data.

In order to improve the extraction of temporal and spatial features from the P300 signal, we designed a CNN network named STCNN with a simple structure in this study. Its detailed structure of feature extractor is shown in Figure 5, the network includes 4 layers, labeled L1–L4.

L1——Input Layer: This layer was applied to load the P300 signal (1 × 150 × 10).

L2——Spatial Convolution Layer: It consists of a convolution kernel of size 10, equal to the number of electrodes. This processing technique uses common space filtering and weighted superposition averaging. The S/N of the signal can be enhanced effectively while the spatial information of redundancy is further removed (Wang et al., 2021). The calculation process is as follows:


$$x^{2}=f\left(I*k^{2}+b^{2}\right)$$

where I is the input data, b is the additive bias, k is the second layer convolution kernel function, and f is the activation function (here is tanh).

L3——Temporal Convolution Layer: It consists of a convolutional kernel of size 4, which can effectively extract the temporal features from the P300 signal. The whole process can be expressed as:


$$x^{3}=f\left(x^{2}*k^{3}+b^{3}\right)$$

As same as L2, Tanh is also applied as the activation function here.

L4——Feature Pooling Layer: Filter the superior features from the features obtained from L3 by the pooling operation. The pooling filter size used in this study is (2, 1). It contributes to reduce the computational complexity and to prevent overfitting with a small number of training samples.

In order to reduce network training time and model complexity, the label classifier of STCNN and the domain discriminator in this study both use the fully connected network, which can also obtain better classification results under the condition of matching with the feature extractor.

## 3. Results

### 3.1. The results in healthy subjects

To verify the validity of the healthy subjects’ data, the most commonly used algorithm (SVM) as well as 3 CNN algorithms (EEGNet, SepConv1D, STCNN) were employed to compare with the proposed WD-ADSTCN for the P300 cross-subject detection in 8 healthy subjects. As shown in Table 2, SVM is difficult to achieve effective results on cross-subject, with an average accuracy of only 64%. In contrast, the deep learning methods have an accuracy of over 70% except for individual subjects. The highest average accuracy of 75% was achieved by STCNN. The average accuracy of EEGNet was 73%. And the average accuracy of SepConv1D was only 72%, which was slightly lower than the other two deep learning methods. The cross-subject average accuracy of WD-ADSTCN on healthy subjects can reach 78%. A one-way repeated measures ANOVA showed that these classification algorithms had significant different results ( $p<10^{-6},F\left(4,35\right)=20.42$ ). Furthermore, the *post-hoc* ANOVA (Bonferroni-corrected) indicated that the average accuracy was significantly higher for the WD-ADSTCN than that for all other methods except STCNN (*p* < 0.05 corrected).

**Table 2:** The cross-subject accuracy comparison in healthy subjects.

Method	H1	H2	H3	H4	H5	H6	H7	H8	Mean	*p*-Value (corrected)
SVM	0.67	0.58	0.65	0.61	0.66	0.61	0.67	0.69	0.64	3.58 x 10^–9^
EEGNet	0.72	0.70	0.72	0.71	0.71	0.76	0.75	0.78	0.73	0.04
SepConv1D	0.70	0.67	0.70	0.70	0.76	0.74	0.74	0.76	0.72	0.01
STCNN	0.73	0.70	0.76	0.72	0.77	0.75	0.76	0.80	0.75	0.51
WD-ADSTCN	0.79	0.75	0.76	0.74	0.78	0.80	0.82	0.81	0.78	–

### 3.2. The results in DOC patients

Patients were divided into two groups according to their improvement in CRS-R scores before and after the trial for 3 months: Group A were patients who did not improve significantly (P1, P2, P3, P4); group B were patients who improved significantly (P5, P6, P7, P8, P9, P10, P11). As shown in Tables 3, 4, the proposed algorithm was compared with the traditional SVM (within-subject and cross-subject) and other two cross-subject deep learning algorithms (EEGNet and SepConv1D). For patients in the group A, the accuracy in each algorithm was below 60% in each algorithm. For patients in the group B, the proposed algorithm and SVM-within achieved an average accuracy higher than 70%, while the accuracies in other algorithms were below 60%. We conducted a one-way repeated measures ANOVA on groups A and B separately, and found that there was no significant difference in results of the classification algorithms within group A ( p>0.05,F(4,16)=0.66 ), while a significant difference in that within group B ( $p<10^{-6},F\left(4,30\right)=50.51$ ). Furthermore, the *post-hoc* ANOVA (Bonferroni-corrected) indicated that the average accuracy was significantly higher for the WD-ADSTCN in group B than for all other methods except SVM-within (*p* < 0.05 corrected).

**Table 3:** The accuracy comparison of P300 detection in group A.

Method	P1	P2	P3	P4	Mean
SVM-within	0.46	0.56	0.58	0.54	0.54
SVM-cross	0.51	0.48	0.52	0.53	0.51
EEGNet	0.45	0.50	0.56	0.48	0.50
SepConv1D	0.55	0.49	0.50	0.47	0.50
WD-ADSTCN	0.52	0.53	0.53	0.50	0.52

**Table 4:** The accuracy comparison of P300 detection in group B.

Method	P5	P6	P7	P8	P9	P10	P11	Mean	Value of *p* (corrected)
SVM-within	0.66	0.70	0.66	0.66	0.78	0.78	0.74	0.71	1.00
SVM-cross	0.50	0.47	0.52	0.56	0.48	0.52	0.54	0.51	$4.06\times 10^{-10}$
EEGNet	0.55	0.51	0.56	0.58	0.51	0.49	0.57	0.54	$8.91\times 10^{-9}$
SepConv1D	0.49	0.53	0.57	0.56	0.54	0.56	0.52	0.54	$8.91\times 10^{-9}$
WD-ADSTCN	0.72	0.68	0.67	0.73	0.71	0.76	0.73	0.71	-

**Figure 6:**
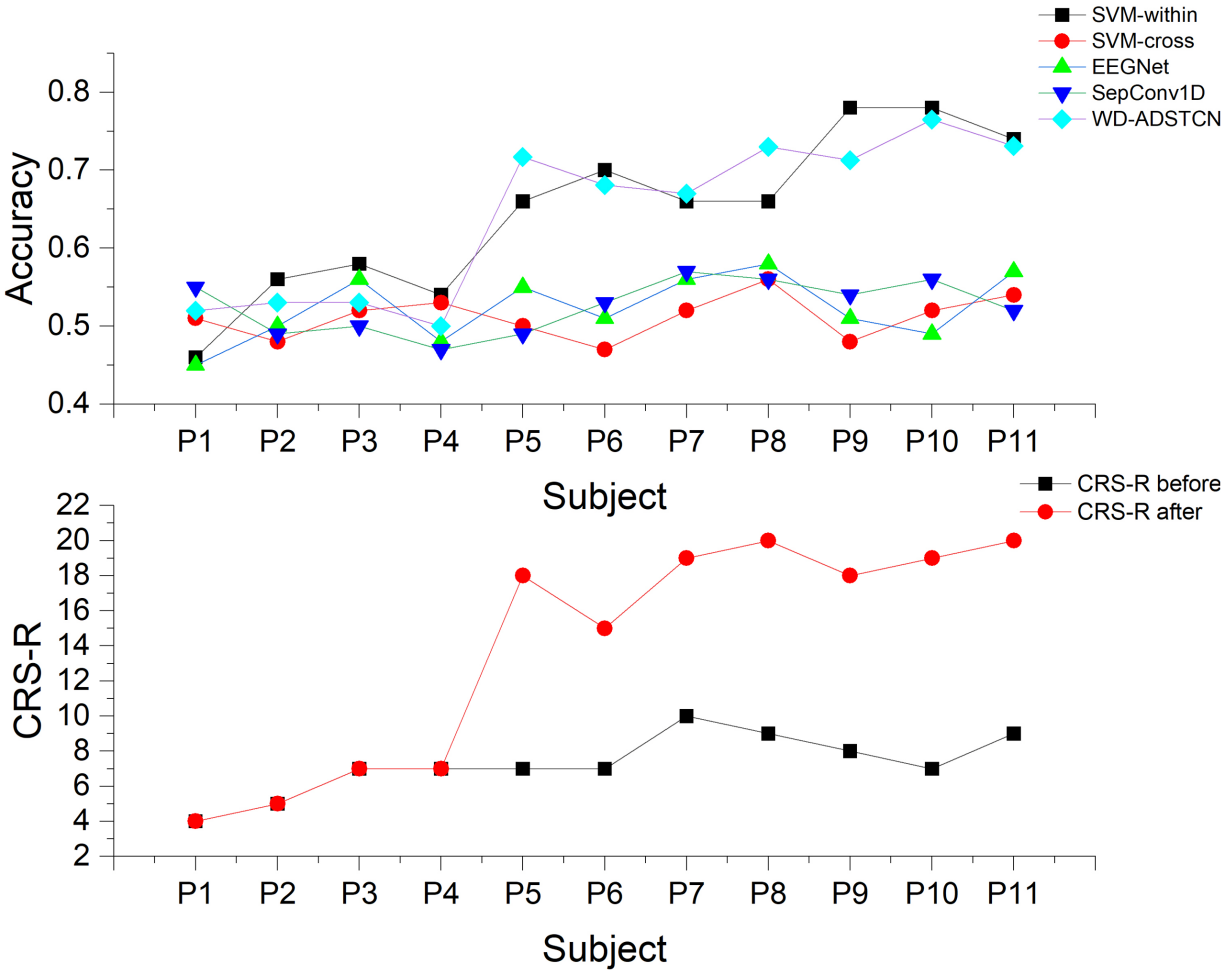
The accuracy and the CRS-R scores in each patient.

To further validate the relationship between P300 detection results and the detection of consciousness in patients with DOC, we compared the accuracy in Table 3 with the CRS-R scores (Table 1) in Figure 6. The accuracy curve of the proposed WD-ADSTCN algorithm is similar to the patients’ CRS-R scores after the experiment. The proposed cross-subject WD-ADSTCN algorithm has achieved similar detection results with that of the within-subject algorithm. Also, the improvement in CRS-R scores of patients (P5, P10) was greater, when WD-ADSTCN accuracy was slightly higher than SVM-within. The results of other three cross-subject methods are different from those of CRS-R after the experiment.

#### 3.2.1. The effect of domain adaptation

To verify the effect of domain adaptation in this study, we designed the following 3 experiments. STCNN_patient: Validate the effect of training STCNN on DOC patient data using a leave-one-out cross-validation; STCNN_healthy_subject: Training STCNN with healthy subjects and testing directly on DOC patients; ADSTCN_patient: Validate the effect of training ADSTCN on DOC patient data using leave-one-out cross-validation.

**Figure 7:**
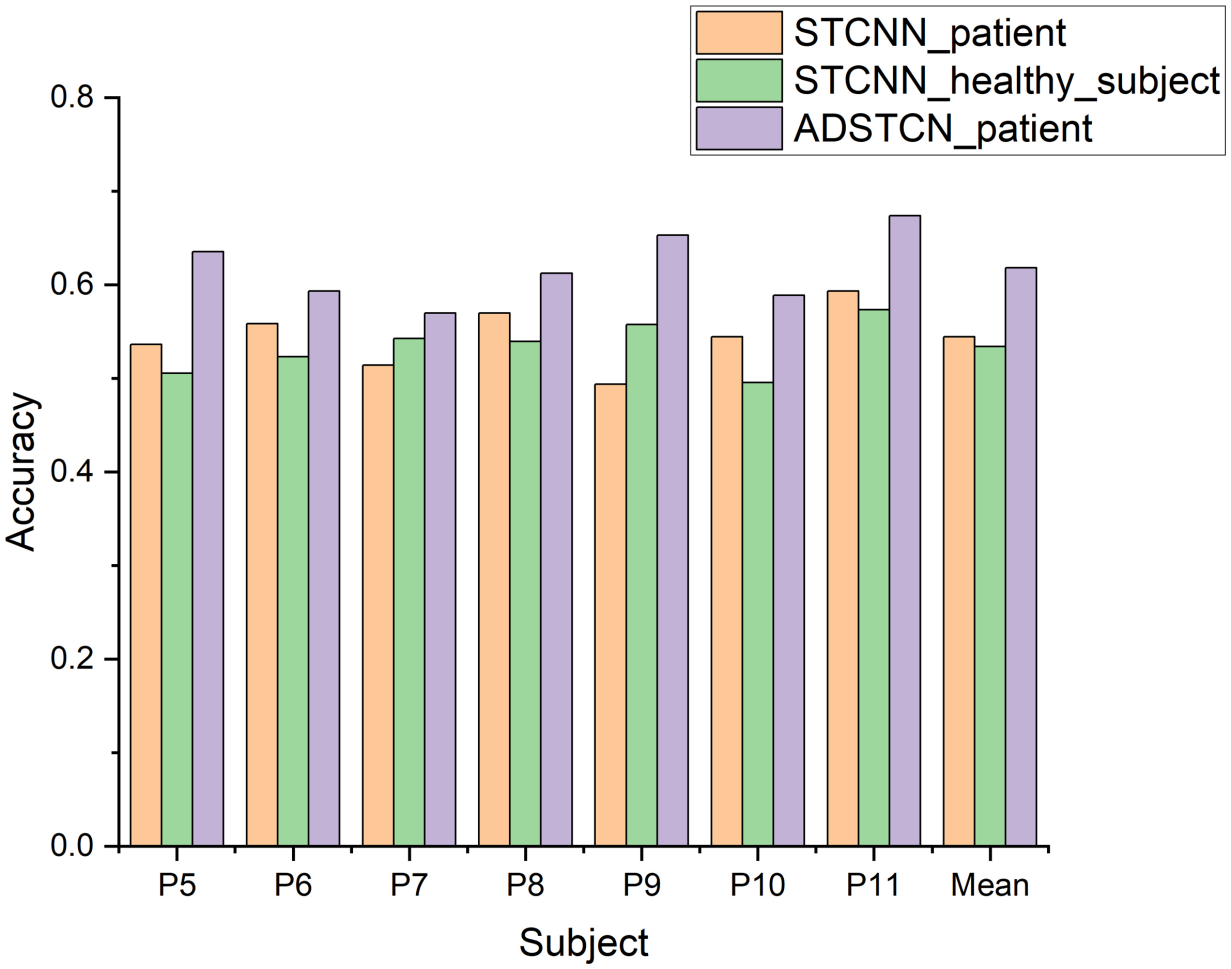
The accuracy of ADSTCN and STCNN for DOC patients in the group B.

As shown in Figure 7, The results of STCNN_patient and STCNN_healthy_subject were extremely unsatisfactory and failed to achieve classification at all (below 64%). Although the average accuracy in ADSTCN_patient was lower than 64%, there were three subjects with good results (P5, P9, P11). The results show that ADSTCN can improve the cross-subject detection accuracy of P300 to some extent, but its enhancement effect is limited under the condition that the subject features are not obvious. Meanwhile, due to the large difference in P300 features between healthy subjects and the DOC patients, the patients could not directly apply the trained model from healthy subjects.

#### 3.2.2. The effect of source domain

To investigate the effect of the source domain on the experimental results, we designed an additional set of experiments to compare with ADSTCN_patient (ADSTCN_healthy_subject: Transfer the healthy subjects training model to DOC patients by using ADSTCN). The source domain in ADSTCN_patient is other DOC patients, while ADSTCN_healthy_subject is healthy subjects.

**Figure 8:**
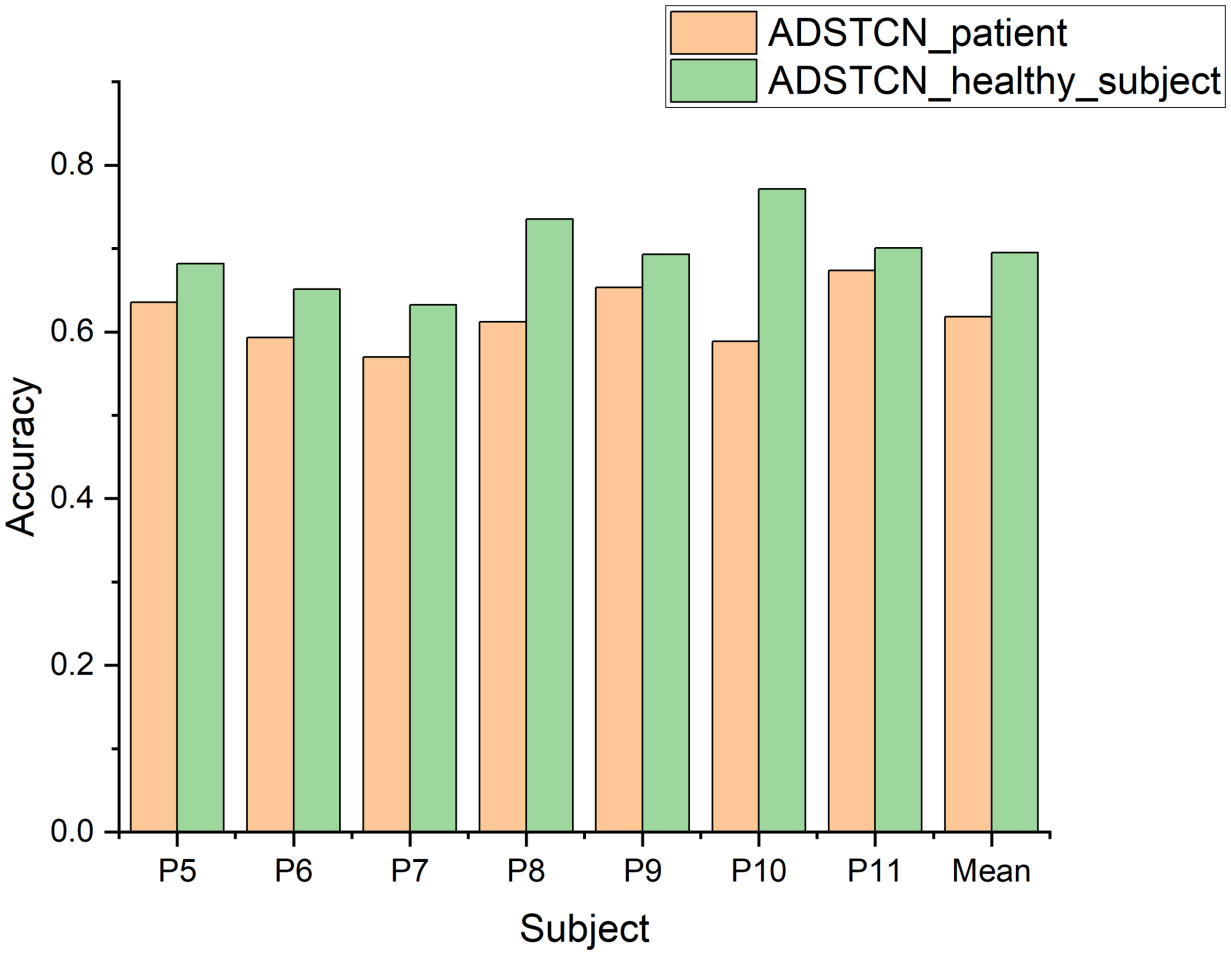
The accuracy of ADSTCN using different source domains for DOC patients in the group B.

The results are shown in Figure 8. The accuracy of using healthy subjects as the source domain was higher than using the other patients as the source domain on all patients. When using healthy subjects as the source domain, multiple patients (P8, P10, P11) had an accuracy rate of over 70%, with an average accuracy of 69.5%.

#### 3.2.3. The effect of subject selection

**Figure 9:**
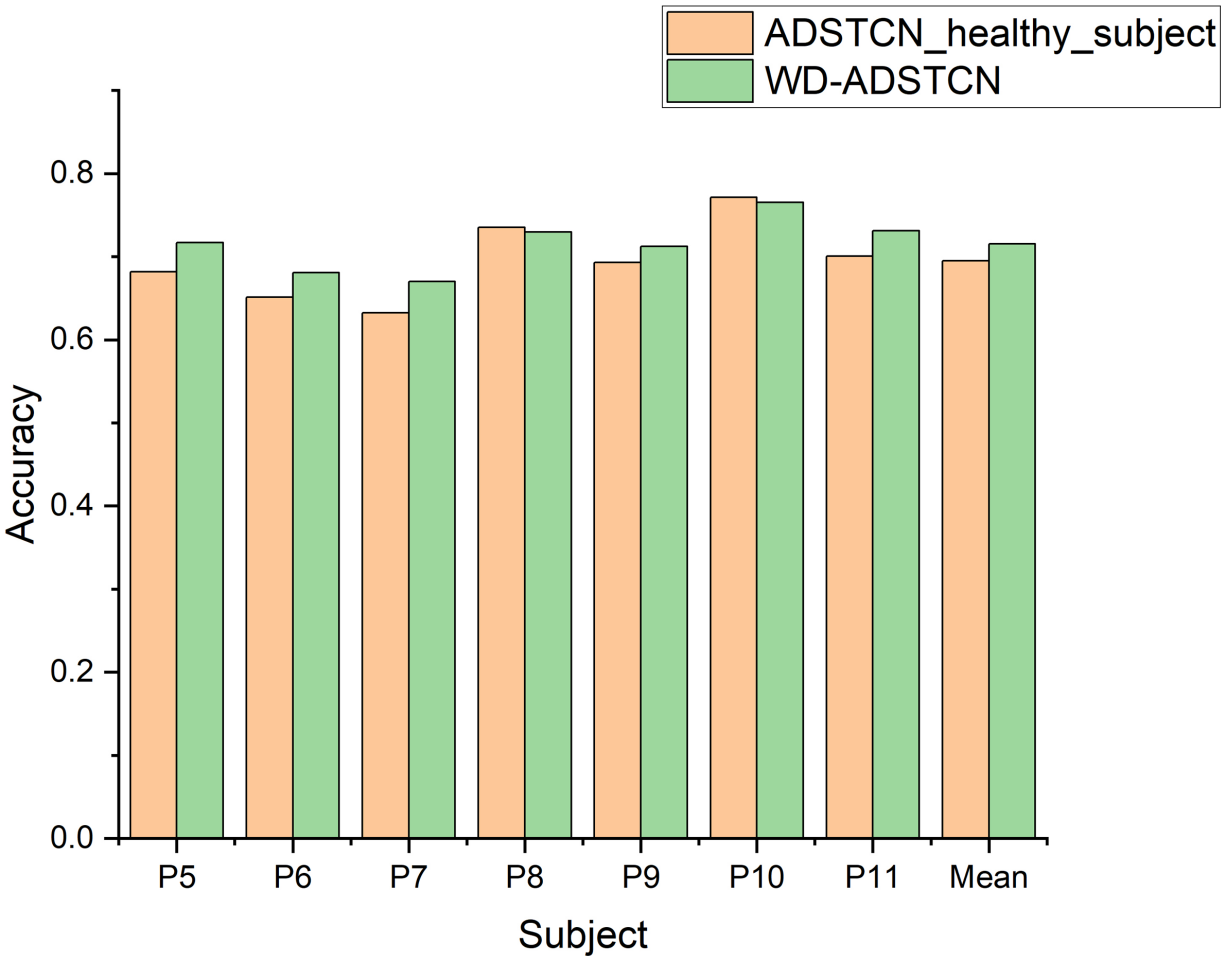
The accuracy of ADSTCN and WD-ADSTCN for DOC patients in the group B.

To confirm the contribution of WD subject selection, we compare the results of ADSTCN_healthy_subject (Without subject selection) with those in Table 3 (WD-ADSTCN). As shown in Figure 9, WD-ADSTCN had an average accuracy of 71.5%, which is better than ADSTCN_healthy_subject. The accuracy of the subjects fluctuated less after using WD, with an improvement in the otherwise poorer subjects (e.g., P5, P6, P7, P11), but a slight decrease in the better subjects (P8, P10). This indicates that WD does remove some of the subjects with side effects, but it may also remove useful ones when the overall effect is better. In this specific condition of DOC patients, WD subject selection has a certain effect.

## 4. Discussion

The BCI-based approach can directly evaluate DOC patients based on brain signals, and this approach may become a clinical tool to assist in the clinical setting. Currently, the researchers are mainly focused on the detection of EEG in DOC patients by using traditional SVM algorithms from intra-subject. This method requires the collection of patients’ data to calibrate the system, which is rather inconvenient in clinical applications. In this regard, this study proposed a method based on a transfer model from healthy subjects to overcome the cross-subject problem of P300 detection in DOC patients. The results of our study show that all 7 patients whose prognosis improved had an accuracy of over 66%. This demonstrates the effectiveness of our algorithm on cross-subject of DOC patients and its possibilities for awareness detection and communication.

Before applicating in the patients, we first verified in the healthy subjects’ data. We compared the proposed WD-ADSTCN algorithm with traditional SVM algorithm and some excellent deep learning networks (EEGNet, SepConv1D) on cross-subject (as shown in Table 2). The results show that the proposed algorithm has indeed some advantages, and demonstrate that the feature extractor of STCNN can extract suitable temporal and spatial features from the P300 signal. Under the condition of simplifying the structure as much as possible, STCNN still has excellent performance.

**Figure 10:**
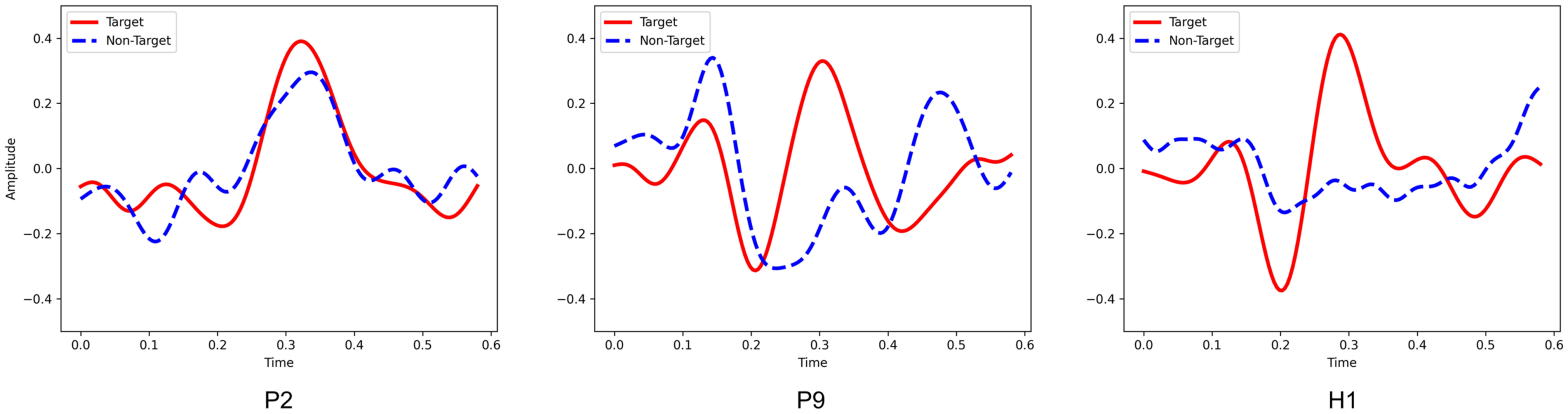
The P300 waveforms from patient P2, patient P9 and healthy subject H1.

The experimental results show that other cross-subject methods (e.g., EEGNet and SepConv1D) performed poor, as shown in Tables 2, 3. Furthermore, the analysis of domain adaptation in Section 3.2.1 found that it is difficult to achieve results either directly in cross-subject on DOC patients or by using the model obtained from healthy subjects on DOC patients. These demonstrated a large difference in the P300 data between DOC patients and healthy subjects, and a similarly large difference between DOC patients. It may be due to the difference in the shifting of the wave peak and amplitude of P300. As shown in Figure 10, for healthy subjects H1, a clear P300 signal typically appears around 300ms after hearing or seeing an unexpected stimulus, with a clear difference in responses between target and non-target stimuli. In contrast, DOC patients do not show so obvious difference in responses as that of healthy subjects. The responses of patient P9 in group B are close to those of healthy subjects, while the target and non-target responses of patient P2 in group A are difficult to distinguish. These results may indicate DOC patients have different abilities to perform selective attention. Patients in group B could selectively pay attention to the target stimuli, but were still unable to completely ignore the non-target stimuli, resulting in some strong non-target responses. This difference may be due to impaired brain function in some DOC patients, leading to a decline in their cognitive ability [3]. In addition, the occurrence rate of P300 in some DOC patients may also decrease. Apart from impaired brain function, this may be due to the loss of consciousness and autonomy, making them unable to consciously respond to external stimuli.

In this study, 7 of 11 patients achieved P300 detection accuracy significantly higher than the chance level (i.e., 64% in Kübler and Birbaumer, 2008), using the proposed WD-ADSTCN algorithm. Furthermore, all the 7 patients showed the improved CRS-R scores after the experiment. These means that the classification accuracy of ADSTCN can be used as a prognostic judgment for DOC patients. Although, the SVM within subject got similar performance. Compared to SVM, ADSTCN can obtain similar results by utilizing easily collected healthy subject’s data with unlabeled patient data. The problem of insufficient DOC patient data is solved to a certain extent.

In the Figure 8, we directly used ADSTCN within patients (other patients as source domain). However, the results have a significant gap compared with from healthy subjects to patients, which may be due to the fact that in the pre-training phase, the mixing of multiple patient data would not be able to get a better classifier, thus making the classifier ineffective even though the features obtained by the feature extractor in the adversarial phase are in the same feature space. And after we joined WD to select subjects, the patients’ overall effect of P300 did improve, but there is a decrease in individual subjects (as shown in Figure 9). Because the calculation of WD is more complicated, it is difficult to use it for selecting individual samples, and we will continue to work on more appropriate sample selection methods from relational networks and distance metric networks.

## 5. Conclusion

The current clinical approach to diagnosing patients with DOC by means of scales has a high rate of misdiagnosis. Although a P300-based BCI system can assist in diagnosis, there is no decoding algorithm that can detect P300 in DOC patients cross-subject. In this study, our proposed ADSTCN algorithm based on domain adaptation can train the initial model using data from the healthy subjects after selection by WD, and then adjusting it to achieve cross-subject effects by using patient data. The results showed that ADSTCN outperformed other methods in cross-subject testing of DOC patients, with the results approaching that of SVM in intra-subject. However, the subject selection module of ADSTCN may filter out useful subjects when the differences between subjects are small which could cause a slight decrease in accuracy. Moreover, the accuracy and stability of BCI technology are still limited due to issues such as signal noise and interference. At the same time, The CRS-R and other assessment scales remain the primary methods for evaluating DOC. Currently, BCI technology can be only served as an auxiliary diagnostic tool (Schnakers, 2020). In the future, we will further improve the subject selection and try to eliminate the differences between healthy subjects and DOC patient groups to obtain a generalized P300 classifier for DOC patients.

## Data availability statement

The original contributions presented in the study are included in the article/supplementary material, further inquiries can be directed to the corresponding author.

## Ethics statement

The studies involving human participants were reviewed and approved by the Ethics Committee of the General Hospital of Guangzhou Military Command. The patients/participants provided their written informed consent to participate in this study.

## Author contributions

FW designed the system, experiment, and paradigm. FQ and FW collected the data. FW, YW, and ZL analyzed the data. FW, YW, and JL wrote the manuscript. All authors contributed to the article and approved the submitted version.

## Funding

This project was funded by the Guangdong Basic and Applied Basic Research Foundation (Grant Nos. 2021A1515011853 and 2021A1515011600), the National Natural Science Foundation of China (Grant Nos. 61906019 and 62006082), STI 2030-Major Projects 2022ZD0208900, Guangzhou Science and Technology Plan Project (Grant No. 202102020877).

## Conflict of interest

The authors declare that the research was conducted in the absence of any commercial or financial relationships that could be construed as a potential conflict of interest.

## Publisher’s note

All claims expressed in this article are solely those of the authors and do not necessarily represent those of their affiliated organizations, or those of the publisher, the editors and the reviewers. Any product that may be evaluated in this article, or claim that may be made by its manufacturer, is not guaranteed or endorsed by the publisher.
